# Educational level and the risk of mental disorders, substance use disorders and self‐harm in different age‐groups: A cohort study covering 1,6 million subjects in the Stockholm region

**DOI:** 10.1002/mpr.1964

**Published:** 2023-02-17

**Authors:** Baojing Li, Peter Allebeck, Bo Burstöm, Anna‐Karin Danielsson, Louisa Degenhardt, Terje A. Eikemo, Alize Ferrari, Ann Kristin Knudsen, Andreas Lundin, Hélio Manhica, John Newton, Harvey Whiteford, Pär Flodin, Hugo Sjöqvist, Emilie E. Agardh

**Affiliations:** ^1^ Department of Public Health Sciences Stockholm University Stockholm Sweden; ^2^ Department of Global Public Health Karolinska Institutet Stockholm Sweden; ^3^ Institute for Health Metrics and Evaluation (IHME) University of Washington Seattle New South Wales USA; ^4^ National Drug and Alcohol Research Centre UNSW Sydney Sydney New South Wales Australia; ^5^ Centre for Global Health Inequalities Research (CHAIN) Department of Sociology and Political Science Norwegian University of Science and Technology (NTNU) Trondheim Norway; ^6^ School of Public Health The University of Queensland Brisbane Queensland Australia; ^7^ Centre for Disease Burden the Norwegian Institute of Public Health Bergen Norway; ^8^ Department of Psychosocial Science University of Bergen Bergen Norway; ^9^ Public Health England (PHE) Health Improvement London UK; ^10^ European Centre for Environment and Health University of Exeter Exeter UK

**Keywords:** educational level, mental disorders, self‐harm, substance use disorders

## Abstract

**Objective:**

To investigate the associations between low education and risk of mental disorders, substance use disorders and self‐harm in different age‐groups.

**Methods:**

All subjects in Stockholm born between 1931 and 1990 were linked to their own or their parent's highest education in 2000 and followed‐up for these disorders in health care registers 2001–2016. Subjects were stratified into four age‐groups: 10–18, 19–27, 28–50, and 51–70 years. Hazard Ratios with 95% Confidence Intervals (CIs) were estimated with Cox proportional hazard models.

**Results:**

Low education increased the risk of substance use disorders and self‐harm in all age‐groups. Males aged 10–18 with low education had increased risks of ADHD and conduct disorders, and females a decreased risk of anorexia, bulimia and autism. Those aged 19–27 years had increased risks of anxiety and depression, and those aged 28–50 had increased risks of all mental disorders except anorexia and bulimia in males with Hazard Ratios ranging from 1.2 (95% CIs 1.0–1.3) for bipolar disorder to 5.4 (95% CIs 5.1–5.7) for drug use disorder. Females aged 51–70 years had increased risks of schizophrenia and autism.

**Conclusion:**

Low education is associated with risk of most mental disorders, substance use disorders and self‐harm in all age‐groups, but especially among those aged 28–50 years.

## 1 INTRODUCTION

The association between socioeconomic position (SEP) and mental disorders has been discussed for many decades (Faris & Dunham, 1939; Goldberg & Morrison, 1963). One explanation for the higher frequency of mental disorders in disadvantaged groups was the concept of “social drift”, that is, that the consequences of mental disorders lead individuals to drift into or fail to rise out of poor socioeconomic conditions (Faris & Dunham, 1939; Goldberg & Morrison, 1963). Emerging evidence, however, show that low SEP is also an important determinant of many mental disorders such as schizophrenia (Lee et al., 2020; Wicks et al., 2010), anxiety and depression (Joinson et al., 2017; Kosidou et al., 2011), substance use disorders (Gauffin et al., 2013; Manhica et al., 2021), and self‐harm (Lodebo et al., 2017). Better understanding of pre‐existing social determinants is important, especially since many proposals to address the major disease burden have focused on improving access to treatment services (Chisholm et al., 2007; World Health Organization, 2019).

Previous longitudinal studies on SEP and mental disorders are commonly based on self‐reported information (Molarius et al., 2009), or representative samples with individuals identified through diagnostic survey interviews (Joinson et al., 2017; Kivimäki et al., 2020). However, these studies are limited by only focusing on one, or a few types of mental disorders at a time, which gives a fragmented picture. Other longitudinal large‐scale studies are based on population‐based hospital registers that are linked to information on socioeconomic factors (Munk‐Jørgensen & Østergaard, 2011). Hospital registers are often comprehensive but have limitations for disorders with very low hospitalization rates such as anxiety, personality disorders, depression and substance use disorders (Munk‐Jørgensen & Østergaard, 2011). In Sweden, as in other high‐income countries, a large proportion of mental disorders among adults are treated only in primary health care (Barbato et al., 2016; Sundquist et al., 2017). The health care system in Sweden has universal coverage and population health care registers are regularly updated (Ludvigsson et al., 2016). One advantage with these registers is that they can be linked with social databases through a unique civic registration number. This enables analysis of SEP and a comprehensive assessment of mental disorders captured in tertiary as well as primary health care over time for total population samples.

At the same time, many mental disorders have an early age of onset (Solmi et al., 2022), but risk to mental disorders manifests themselves at all stages in life (Chen et al., 2018; Lynch et al., 2021; Ramos & Stanely, 2020). Better understanding of social determinants that precede the outcome being studied in different age groups, using longitudinal design is needed (Alegría et al., 2018). This will not only inform the academic debate on causation and selection mechanisms, but also the extent to which the burden of these disorders in society could potentially be prevented (Allen et al., 2014).

Education, income, and occupation are common measures of SEP. Educational attainment usually occurs in early life and has been described as capturing social opportunities and the transition from parental SEP to adult SEP, reflecting factors such as material and intellectual resources of family origin. Educational attainment is also a strong determinant of the individual's future employment and income (Galobardes et al., 2006). Educational attainment, in contrast to occupation and income, thus represents an early determinant of SEP.

### 1.1 Aim of the study

We aimed to investigate the associations between low education and the risk of subsequent mental disorders, substance use disorders and self‐harm in four age‐groups: later childhood and adolescence (10–18 years), young adulthood (19–27 years), mid‐life (28–50 years), and later life (51–70 years). The study is based on all subjects aged 10–70 years in the Stockholm region of Sweden, approximately 1.6 million inhabitants, linked to their educational level at baseline, and followed up for diagnoses in patient registers covering inpatient and outpatient psychiatric care as well as primary care. In order to capture effect of education at early age, we used parental education as exposure for subjects between 10 and 27 years of age at baseline.

## 2 METHODS

### 2.1 Participants

**FIGURE 1:**
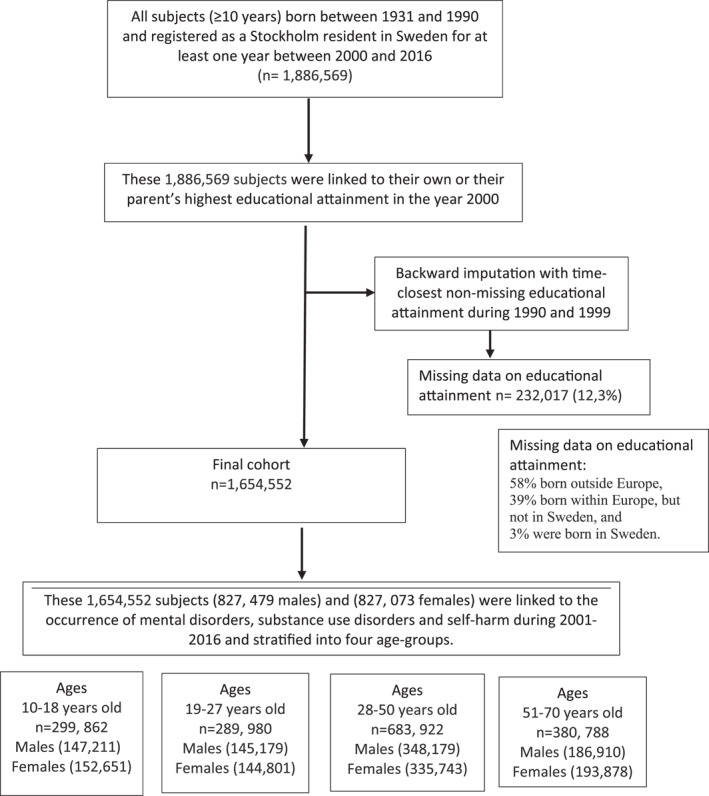
Flow diagram.

This is a cohort study based on the total population in Stockholm Region in Sweden, around 2 M inhabitants. Flow scheme of the subjects included is shown in Figure 1. From the Register of the Total Population, comprising standard demographic information on subjects as well as personal identifiers of their parents, all subjects born between January 1^st^, 1931 and December 31^st^, 1990 who had been registered in the Stockholm Region for at least one year during January 1^st^, 2001 to December 31^st^, 2016 were identified (*N* = 1,886,569). These subjects were linked by their unique civic registration number to data on highest educational attainment between January 1^st^ and December 31^st^ in 2000, that is, when they were 28 years or older. Since subjects aged 10–27 years may not have reached their highest educational level, we used their parents' highest educational level as exposure. We performed a backward imputation with time‐closest non‐missing educational attainment to fill in potential missing information on highest attained educational attainment in the year 2000. We excluded subjects with no information on education (*N* = 232,017, 12,3%). Among those excluded, 58% were born outside Europe, 39% were born within Europe, but not in Sweden, and 3% were born in Sweden. The final cohort consisted of 1,611,881 subjects who were followed between January 1^st^, 2001, and December 31^st^, 2016 for a clinical diagnosis of mental or substance use disorder or self‐harm. We stratified the final cohort into males and females and by four age‐groups, those aged 10–18 years at baseline, 19–27 years, 28–50 years, and 51–71 years.

### 2.2 Exposure

Data on level of educational attainment, both the subject's own, and the parent with highest attained education, was obtained from the Longitudinal Integrated database for Health Insurance and Labor Market Studies (LISA). Educational attainment was classified as high (post‐secondary or tertiary education with more than 12 years of study), middle (upper secondary education with 9–12 years of study), and low (primary or lower secondary education with overall 9 years of study or less) according to the International Standard Classification of Education 1997 (ISCED 97).

### 2.3 Outcome

#### 2.3.1 Assessment of outcome

Data on occurrence of health care episodes was obtained through the Stockholm administrative health care database VAL (Vårdanalysdatabaserna; Stockholm regional health care data warehouse). VAL contains individual data on persons admitted to health care in publicly funded in‐ and outpatient psychiatric care as well as primary health care. Registers available cover in‐patient care for the years 1990 and 2016, and outpatient as well as primary care from 2000, although these two latter with varying degree of diagnostic information prior to the year 2001. Patients are recorded with diagnoses coded according to the International Classification of Diseases (ICD), 10th revision (ICD‐10). Virtually all care in Sweden is publicly funded, although treatment may be performed by private providers. The Region Stockholm database comprises 15 hospitals providing in‐patient care also for psychiatric patients, 14 psychiatric out‐patient care centers, and approximately 200 primary health care centers where many less serious mental disorders are treated.

#### 2.3.2 Clinical diagnoses

Disease classification followed the WHO ICD. The cause groups for mental disorders with corresponding ICD‐10 codes are: *Schizophrenia (*F20‐F20.9, F25‐F25.9), *major depression* (F32‐F33.9), *dysthymia* (F34.1), *bipolar disorder* (F30‐F31.9, F34.0), *anxiety disorders* (F40‐F44.9, F93‐F93.2), *anorexia nervosa* (F50.0‐F50.1), *bulimia nervosa* (F50.2‐F50.5), *autism spectrum disorders* (F84‐F84.9), *ADHD* (F90‐F90.9), and *conduct disorders* (F91‐F92.9). Substance use disorders include the following: *Alcohol use disorder* (F10‐F10.99, G31.2, R78.0, X45‐X45.9, X65‐X65.9, Y15‐Y15.9, Z81.1), *drug use disorder* (F11‐F19.99, P96.1, R78.1‐R78.9, Z81.2‐Z81.4); and *self‐harm* (X60‐X64.9, X66‐X84.9, Y87.0). When appropriate, that is, when accounting for diagnoses going back in time, such as parental diagnoses or diagnoses a couple of years before baseline we used corresponding ICD‐9 codes. We included number of unique diagnoses for each individual, where the same diagnosis was not counted more than once per person at follow‐up, but the same person could, however, contribute to more than one diagnosis.

### 2.4 Statistical analysis

First, the hazard proportion assumption was assessed, using a graphical examination, the Kaplan‐Meier, to investigate whether education was approximately constant over time for each outcome (showing a proportional effect). Second, Hazard Ratios (HRs) with 95% confidence intervals (CIs) were estimated for low and middle educational level in 2000 and the risk of each unique mental disorder, substance use disorder and self‐harm during 2001 and 2016 for each age‐stratum, in separate Cox proportional hazard models. Each unique diagnose was based on date of first diagnosis during follow‐up, and those diagnosed with the same condition up to 10 years prior to 2001 were excluded in each separate model, with the intention to reduce the risk of reverse causality. We used age as the time scale, which was based on birth year in 5‐year intervals within each age stratum, to adjust for potential heterogeneity over birth year. Subjects who were not diagnosed with any of these conditions during 2001 through 2016 were censored based on death date, recorded date of moving out of the Stockholm Region, or December 31^st^, 2016, which ever came first. We adjusted for life‐time biological parental mental disorders, to account for any genetic liability using the same registers. If at least one of the biological parents had a mental diagnosis we classified this covariate as yes, if neither of them had a diagnosis we classified it as no. We used SAS version 9.4 software for statistical analysis.

## 3 RESULTS

In the final cohort of 1,654,552 subjects (50% males and 50% females), the majority of males (Table 1) and females (Table 2) had high or middle educational attainment. Schizophrenia, major depression, anxiety, ADHD, conduct disorders, alcohol and drug use disorder, and self‐harm were more common among those with lower compared to those with high educational attainment in both males (Table 1) and females (Table 2). The occurrence of dysthymia, bipolar disorder, anorexia, bulimia, and autism spectrum disorder did not differ to any important extent across educational groups.

**TABLE 1:** Characteristics of males with a first diagnosis of mental disorder, substance use disorders and self‐harm during 2001–2016 and highest educational attainment in 2000.

Males	Educational levels			
	Total	High	Middle	Low
*n* (%)	827,479	356,008 (43%)	345,652 (42%)	125,819 (15%)
Clinical diagnoses	*n* ^a^ %^b^	*n* ^a^ %^b^	*n* ^a^ %^b^	*n* ^a^ %^b^
Schizophrenia	3804 0,5	1053 0,3	1704 0,5	1048 0,8
Major depression	76,168 9,0	29,422 8,2	33,870 9,8	12,876 10,0
Dysthymia	2656 0,3	1079 0,3	1200 0,3	397 0,3
Bipolar disorder	6115 0,7	2671 0,8	2611 0,8	833 0,7
Anxiety disorders	106,859 13,0	41,560 11,7	27,155 7,9	17,144 13,6
Anorexia nervosa	95 0,01	46 0,01	34 0,01	15 0,01
Bulimia nervosa	143 0,02	55 0,02	67 0,02	21 0,01
Autism spectrum dis	4778 0,6	2080 0,6	2039 0,6	659 0,5
ADHD	11,609 1,4	3958 1,1	5723 1,7	1928 1,5
Conduct disorders	655 0,08	213 0,05	310 0,09	132 0,1
Alcohol use disorder	42,465 5,1	11,846 3,2	20,766 6,0	9853 7,8
Drug use disorder	26,722 3,2	6489 1,8	13,028 3,8	7205 5,7
Self‐harm	3036 0,4	826 0,2	1519 0,4	691 0,5

^a^ Occurrence, that is, each diagnose is only present once, first time diagnosis for each condition, although the same person can be diagnosed with several conditions.

^b^ Percentage of the diagnose at each educational level (among all participants at the same educational level).

**TABLE 2:** Characteristics of females with a first diagnosis of mental disorder, substance use disorders and self‐harm during 2001–2016 and highest educational attainment in 2000.

Females	Educational levels			
	Total	High	Middle	Low
*n* (%)	827,073	364,864 (44%)	348,136 (42%)	114,073 (14%)
Clinical diagnoses	*n* ^a^ %^b^	*n* ^a^ %^b^	*n* ^a^ %^b^	*n* ^a^ %^b^
Schizophrenia	3339 0,4	974 0,2	1529 0,4	1536 1,3
Major depression	137,565 16,6	55,832 15,0	60,916 17,5	20,817 18,0
Dysthymia	3554 0,4	1432 0,4	1603 0,5	515 0,5
Bipolar disorder	9612 1,2	4222 1,2	4246 1,2	1144 1,0
Anxiety disorders	211,530 25,6	88,538 24,3	94,121 27,0	28,871 25,0
Anorexia nervosa	1756 0,2	990 0,3	639 0,2	127 0,1
Bulimia nervosa	2529 0,3	1233 0,3	1072 0,3	224 0,2
Autism spectrum dis	3282 0,4	1369 0,4	1488 0,4	425 0,4
ADHD	10,295 1,2	3727 1,0	5073 1,5	1495 1,3
Conduct disorders	625 0,08	233 0,06	272 0,07	120 0,1
Alcohol use disorder	33,578 4,1	7628 2,0	11,759 3,4	4192 3,7
Drug use disorder	25,151 3,0	6402 1,8	19,228 5,5	5923 5,2
Self‐harm	4226 0,5	1436 0,4	2027 0,6	763 0,7

^a^ Occurrence, that is, each diagnose is only present once, first time diagnosis for each condition, although the same person can be diagnosed with several conditions.

^b^ Percentage of the diagnose at each educational level (among all participants at the same educational level).

Tables 3 and 4 shows the risks of different mental disorders, substance use disorders and self‐harm in different age groups, by level of education. Full data on number of subjects in different age‐groups are shown in appendix Tables 1 and 2. Lower educational attainment was associated with increased risks of alcohol‐ and drug use disorders and self‐harm in all age‐groups in both males (Table 3) and females (Table 4).

**TABLE 3:** Hazard Ratios (HRs) (HR) with 95% Confidence Intervals (CIs) for the association between highest attained education at baseline (in 2000) and risk of mental disorders, substance use disorders and self‐harm during follow‐up (2001–2016) in four different age groups among males.

Clinical diagnoses	10–18 years at baseline			19–27 years at baseline			28–50 years at baseline			51–70 years at baseline		
	Educational levels (parental)			Educational levels (parental)			Educational levels			Educational levels		
	High	Middle	Low	High	Middle	Low	High	Middle	Low	High	Middle	Low
Schizophrenia	1.0	1.0 (0.8–1.2)	1.3 (1.0–1.8)	1.0	0.9 (0.7–1.1)	1.8 (1.4–2.2)	1.0	2.4 (2.1–2.7)	4.2 (3.7–4.8)	1.0	1.3 (1.1–1.6)	2.1 (1.7–2.5)
Major depression	1.0	1.0 (1.0–1.1)	1.0 (0.9–1,0)	1.0	1.1 (1.1–1.1)	1.2 (1.1–1.2)	1.0	1.3 (1.3–1.3)	1.6 (1.5–1.6)	1.0	1.0 (1.0–1.1)	1.0 (1.0–1.0)
Dysthymia	1.0	0.9 (0.8–1.1)	0.7 (0.5–1.0)	1.0	1.0 (0.8–1.2)	0.9 (0.7–1.3)	1.0	1.3 (1.1–1.4)	1.5 (1.3–1.7)	1.0	1.0 (0.8–1.3)	0.8 (0.6–1.1)
Bipolar disorder	1.0	0.8 (0.7–1.0)	0.7 (0.5–0.9)	1.0	0.9 (0.8–1.0)	1.0 (0.9–1.3)	1.0	1.2 (1.1–3.0)	1.2 (1.0–1.3)	1.0	0.7 (0.6–0.9)	0.6 (0.5–0.7)
Anxiety dis	1.0	1.1 (1.1–1.1)	1.1 (1.1–1.2)	1.0	1.2 (1.1–1.2)	1.3 (1.2–1.3)	1.0	1.3 (1.3–1.3)	1.5 (1.5–1.6)	1.0	1.1 (1.1–1.2)	1.1 (1.1–0.2)
Anorexia	1.0	0.8 (0.4–1.4)	0.6 (0.2–2.1)	1.0	0.7 (0.3–2.0)	1.9 (0.6–6.1)	1.0	1.5 (0.4–6.5)	3.5 (0.8–16)	1.0	1.0 (0.2–4.6)	1.2 (0.2–6.2)
Bulimia	1.0	1.6 (0.9–2.9)	0.9 (0.3–3.2)	1.0	0.6 (0.3–1.4)	1.9 (0.8–4.6)	1.0	2.3 (1.1–4.6)	1.8 (0.7–4.7)	1.0	0.4 (0.0–2.1)	0.8 (0.2–3.8)
Autism	1.0	0.9 (0.8–1.0)	0.7 (0.6–0.8)	1.0	1.0 (0.9–1.1)	0.9 (0.7–1.1)	1.0	1.3 (1.2–1.5)	2.6 (2.3–3.0)	1.0	1.0 (0.7–1.6)	1.1 (0.7–1.9)
ADHD	1.0	1.3 (1.2–1.4)	1.2 (1.1–1.3)	1.0	1.4 (1.3–1.5)	1.6 (1.4–1.8)	1.0	2.3 (2.1–2.5)	4.0 (3.6–4.4)	1.0	1.1 (0.7–1.5)	1.1 (0.7–1.7)
Conduct dis	1.0	1.5 (1.1–1.9)	1.6 (1.1–2.5)	1.0	1.3 (0.9–1.9)	1.1 (0.6–2.1)	1.0	2.0 (1.4–2.9)	4.2 (2.9–6.2)	1.0	1.3 (0.8–2.1)	1.5 (0.9–2.6)
Alcohol use dis	1.0	1.4 (1.3–1.4)	1.5 (1.3–1.6)	1.0	1.5 (1.4–1.6)	1.8 (1.7–2.0)	1.0	2.2 (2.1–2.3)	3.2 (3.0–3.3)	1.0	1.6 (1.5–1.6)	1.9 (1.8–2.0)
Drug use dis	1.0	1.6 (1.5–1.7)	2.2 (2.0–2.4)	1.0	1.7 (1.6–1.8)	2.5 (2.2–2.7)	1.0	2.8 (2.7–3.0)	5.4 (5.1–5.7)	1.0	1.8 (1.7–1.9)	2.4 (2.3–2.6)
Self‐harm	1.0	1.5 (1.3–1.8)	1.7 (1.3–2.1)	1.0	1.7 (1.4–2.0)	2.3 (1.8–2.9)	1.0	2.6 (2.3–3.1)	5.0 (4.2–5.9)	1.0	1.6 (1.3–2.1)	1.7 (1.3–2.3)

*Note*: All analyses are adjusted for life‐time biological parental mental disorders.

**TABLE 4:** Hazard Ratios (HRs) (HR) with 95% Confidence Intervals (CIs) for the association between highest attained education at baseline (in 2000) and risk of mental disorders, substance use disorders and self‐harm during follow‐up (2001–2016) in four different age groups among females.

Clinical diagnoses	10–18 years at baseline			19–27 years at baseline			28–50 years at baseline			51–70 years at baseline		
	Educational levels (parental)			Educational levels (parental)			Educational levels			Educational levels		
	High	Middle	Low	High	Middle	Low	High	Middle	Low	High	Middle	Low
Schizophrenia	1.0	1.0 (0.8–1.2)	1.2 (0.8–1.8)	1.0	1.1 (0.8–1.4)	1.2 (0.8–1.7)	1.0	1.8 (1.6–2.0)	3.3 (2.9–3.8)	1.0	1.6 (1.4–1.9)	2.1 (1.8–2.5)
Major depression	1.0	1.1 (1.0–1.1)	1.0 (0.9–1.0)	1.0	1.1 (1.1–1.2)	1.2 (1.1–1.2)	1.0	1.2 (1.1–1.2)	1.5 (1.5–1.6)	1.0	1.1 (1.0–1.1)	1.0 (1.0–1.1)
Dysthymia	1.0	0.9 (0.8–1.1)	0.8 (0.6–1.0)	1.0	1.1 (0.9–1.3)	1.1 (0.8–1.4)	1.0	1.4 (1.3–1.6)	1.9 (1.7–2.2)	1.0	0.9 (0.7–1.1)	0.7 (0.6–1.0)
Bipolar disorder	1.0	1.0 (0.9–1.2)	0.7 (0.6–0.9)	1.0	1.1 (0.8–1.0)	1.0 (0.9–1.3)	1.0	1.1 (1.0–1.2)	1.3 (1.2–1.5)	1.0	0.8 (0.7–0.9)	0.6 (0.5–0.7)
Anxiety dis	1.0	1.1 (1.1–1.1)	1.0 (1.0–1.1)	1.0	1.1 (1.1–1.1)	1.2 (1.3–1.2)	1.0	1.2 (1.1–1.2)	1.3 (1.3–1.4)	1.0	1.1 (1.0–1.1)	1.0 (1.0–1.1)
Anorexia nervo	1.0	0.6 (0.5–0.7)	0.4 (0.3–0.5)	1.0	0.8 (0.6–1.0)	0.9 (0.6–1.2)	1.0	1.2 (0.9–1.6)	1.8 (1.3–2.7)	1.0	1.1 (0.6–2.1)	0.8 (0.3–1.9)
Bulimia nervosa	1.0	0.8 (0.7–0.9)	0.6 (0.4–0.7)	1.0	1.3 (1.1–1.5)	1.2 (0.9–1.5)	1.0	1.4 (1.2–1.7)	1.8 (1.4–2.4)	1.0	0.8 (0.4–1.6)	0.3 (0.1–1.1)
Autism	1.0	1.1 (0.9–1.2)	0.8 (0.6–0.9)	1.0	1.0 (0.9–1.2)	1.0 (0.8–1.2)	1.0	1.5 (1.3–1.8)	2.9 (2.5–3.5)	1.0	1.3 (0.7–2.4)	3.0 (1.6–5.5)
ADHD	1.0	1.3 (1.2–1.5)	1.2 (1.1–1.4)	1.0	1.4 81.2–1.5)	1.4 (1.2–1.6)	1.0	1.8 (1.7–2.0)	3.3 (3.0–3.7)	1.0	0.9 (0.6–1.3)	0.6 (0.4–1.1)
Conduct dis	1.0	1.1 (0.8–1.5)	1.4 (0.9–2.2)	1.0	1.0 (0.6–1.6)	1.3 (0.6–2.5)	1.0	1.7 (1.2–2.3)	3.4 (2.3–5.0)	1.0	1.0 (0.6–1.6)	1.4 (0.8–2.3)
Alcohol use dis	1.0	1.4 (1.3–1.4)	1.3 (1.2–1.4)	1.0	1.5 (1.4–1.6)	1.5 (1.3–1.7)	1.0	1.9 (1.8–2.0)	2.6 (2.5–2.8)	1.0	1.5 (1.4–1.6)	1.5 (1.4–1.7)
Drug use dis	1.0	1.6 (1.5–1.7)	1.8 (1.6–2.0)	1.0	1.7 (1.6–1.9)	2.2 (1.9–2.4)	1.0	2.4 (2.4–2.6)	4.2 (4.0–4.5)	1.0	1.8 (1.7–1.9)	2.1 (1.9–2.2)
Self‐harm	1.0	1.2 (1.1–1.3)	1.5 (1.2–1.7)	1.0	1.6 (1.3–1.8)	1.8 (1.5–2.3)	1.0	2.1 (1.8–2.4)	3.9 (3.3–4.5)	1.0	1.2 (1.0–1.5)	1.2 (1.0–1.6)

*Note*: All analyses are adjusted for life‐time biological parental mental disorders.

In later childhood and adolescence, when subjects were 10–18 years old, lower parental educational attainment increased the risk of schizophrenia, ADHD and conduct disorders in males (Table 3), but not obviously in females (Table 4). On the other hand, lower parental educational attainment decreased the risk of anorexia, bulimia and autism in females, and bipolar disorder and autism in males.

In young adulthood (ages 19–27 years), increased risks were found for ADHD, major depression and anxiety disorders in males and females, schizophrenia and conduct disorders in males and bulimia in females. No associations were found for dysthymia, bipolar disorder, anorexia, bulimia and autism in males and females or conduct disorders in females.

In midlife, when subjects were 28–50 years old, lower educational attainment increased the risk of all mental disorders, except anorexia and bulimia in males.

In later life, when subjects were 51–70 years of age, lower educational attainment increased the risk of schizophrenia in males (Table 3) and females (Table 4), and autism in females, and in contrast to those in mid‐life having increased risks for most mental disorders, no other associations were found at this stage in life. Nearly all results showed gradually increased risks the lower the educational level for most outcomes. Adjusting for life‐time parental mental disorders did not influence these results to any important extent (crude data not shown).

## 4 DISCUSSION

Our study shows that low educational attainment is associated with a range of mental disorders, substance use disorders and self‐harm in both males and females. Increased risks were found in all age‐groups, to a varying extent. Most pronounced was the increased risk of alcohol and drug use disorders, and self‐harm, among those with lower educational attainment in all age‐groups, as well as the increased risks of most mental disorders in subjects aged 28–50 years old. There was an increased risk only for schizophrenia, and autism in females aged 51–70 years. In the youngest age groups, low parental educational attainment increased the risk of ADHD and conduct disorders in males, and decreased the risk of anorexia, bulimia and autism in females, and bipolar disorder and autism in males.

Our results are in line with previous cohort studies showing that low SEP is associated with increased risk of schizophrenia (Lee et al., 2020; Wicks et al., 2010), anxiety and depression (Joinson et al., 2017; Kosidou et al., 2011; Stansfeld et al., 2011) ADHD (Russell et al., 2016), substance use disorders (Gauffin et al., 2013; Manhica et al., 2021), and self‐harm (Lodebo et al., 2017). In our study, the risk of schizophrenia increased at lower level of attained education in all age‐groups for males, and in mid‐life and later life in females. Due to the youth preponderance of schizophrenia, with peak age of onset around 20 years (Solmi et al., 2022), prior research has mainly focused on early risk factors, and less on late onset cases (Chen et al., 2018). Older age of onset has however, in contrast to our findings been associated with higher educational achievement. At the same time, unemployment has been associated with the onset of later life schizophrenia (Chen et al., 2018) suggesting the importance of social determinants at a later stage in life as well. On the other hand, the risk of depression and anxiety disappeared in later life in our study. This is somewhat surprising considering the close link between SEP and the risk of these disorders (Kosidou et al., 2011; Stansfeld et al., 2011), and the onset of many new cases in later life.

In line with our findings, a recent systematic review and meta‐analysis based on studies across five continents showed, that children in families of low SEP are more likely to have ADHD that their peers in high SEP families (Russell et al., 2016). However, since ADHD usually manifests in early life (Solmi et al., 2022) the increased risks of ADHD due to lower educational attainment among those aged 28–50 years in our study, suggest a late recognition if this disorder in health care, and implies a two‐way relationship between lower educational attainment and ADHD over the life course.

The mechanisms through which low educational attainment increases the risk of these disorders cannot be derived from this study. Low education is closely related to more disadvantaged living conditions for example, poorer housing conditions, or poorer work conditions, which thus may directly affect mental health, through increased stress (Allen et al., 2014). Chronic stress may affect the hypothalamus‐pituitary‐adrenal (HPA) axis function and cause changes in the neuroendocrine system with potential consequences for the manifestation of psychotic, mood, anxiety and depressive symptoms (Morris et al., 2019; Stephens & Wand, 2012). It is also possible that genetic components and vulnerability may play a role (Lee et al., 2020). In our study, parental mental conditions did however, not explain our results, indicating the possibility of a causal effect of low education. This is in line with previous research, for example, that children in families with disadvantaged socioeconomic circumstances had increased risk for psychosis, regardless of biological parental history of psychosis (Wicks et al., 2010). Similarly, we previously found that exposure to poverty in early life increased the risk of drug use disorders in adolescence, irrespective of parental psychiatric conditions (Manhica et al., 2021).

Appropriate health care and social services are essential to address and alleviate the consequences of mental disorders, and in many countries' treatment coverage is not adequate (World Health Organization, 2019). Moreover, psychiatric co‐morbidity is common, with known problems for health and social services (Achim et al., 2011). The focus of our study however, on the possible contributing role of lower educational attainment and the risk of each mental disorder in different age‐groups, points to the importance of also addressing the underlying social determinants to help reduce the burden of these disorders. Our results may also have implications for research. To date, the influential Global Burden of Disease (GBD) study has not included individual SEP as a risk factor. The GBD is the leading global information system for tracking and comparing disease burden at the global, national and sub‐national level, and results are often used for policy decisions. The burden attributed to risk factors in GBD is based on risk‐outcome pairs, and it is a challenge to include education as a risk factor given the assumption that relative risks are generalizable across populations (Stanaway et al., 2018). Potential challenges can be overcome if estimates within the GBD system become more contextualized. In this study we used the same disease definitions as those in GBD to help inform the GBD study; our estimates can be generalized to high‐income countries.

### 4.1 Methodological considerations

There are some methodological issues that may influence our results. First, many mental disorders have an early age of onset, although treatment typically occurs a number of years later. We excluded from each model those diagnosed with the same condition up to 10 years prior to follow‐up but cannot exclude the possibility that cases are not recurrent cases, or that subjects with early psychological symptoms have not received proper care, or that untreated disorders could have impacted on education, which could imply reversed causality. To assess whether subjects with a specific diagnosis may have had other mental disorders interfering with education, we performed a sensitivity analysis by excluding those with any of the targeted outcome disorder, that is, mental disorder, substance use disorder or self‐harm. In overall, there were some smaller changes in HRs and CIs, although not to the extent that it had any crucial influence on the interpretation of our results. Four estimates were, however, no longer statistically significant, that is, in males aged 10–18 years with low education and schizophrenia, and males aged 28 to 50 with bipolar disorder, as well as in females aged 28–50 years with low education and anorexia and females aged 51–70 years with self‐harm (data not shown). It is important to acknowledge; the quality in the in‐patient specialized care register is regarded as high between 1990 and 2016, but this is not the case for the outpatient and primary care prior to the year 2001. Moreover, subjects who immigrated to Stockholm prior to that will most certainly not have their mental history registered there. Thus, the “wash‐out” effect of excluding prior diagnoses should be interpreted with caution.

Second, even if we adjusted for parental diagnoses, it is possible that other associated risk factors could play a role such as bullying victimization, intimate partner violence and childhood sexual abuse. Lacking such data availability, we were not able to adjust for or otherwise investigate these risk factors in our model. Such factors might also be mediating factors between low educational level and mental health problems, and their role would be interesting to analyze separately.

Third, we excluded 12.3% of the population since we lacked data on attained education. The majority of these were born outside Europe or within Europe but outside Sweden. Depending on their attained education and diagnose status may lead to either an overestimation or underestimation of the associations observed.

Fourth, detecting cases through health care register reflects health‐seeking behavior or utilization of health care. In Sweden, utilization of care for many mental disorders, such as for example, depression and anxiety, has increased since 2007, and it has been suggested that those with lower SEP seeks mental health care, to a lower extent than those with higher SEP (Forslund et al., 2020). If anything, this would lead to an underestimation of the true association between lower attained educational level and these disorders.

A major strength of our study is the use of data from both secondary and primary health care that was linked by a unique identifier to educational level, covering a large population. The universal health coverage in Sweden also ensures high coverage of our data on clinical diagnoses in health care. Our individual data also allowed us to ensure that there were no overlaps or double counting in health care visits over the study period.

### 4.2 Conclusion

Lower attained education seems to be an important determinant of most mental disorders, substance use disorders and self‐harm, and with increased risks varying by age‐groups. The heaviest burden was found in mid‐life. Considering the longitudinal and population‐based design, our results add to previous findings and may help inform policy on the role of social determinants for mental health.

## CONFLICTS OF INTEREST STATEMENT

None.

## ETHICS STATEMENT

The authors assert that all procedures contributing to this work comply with the ethical standards of the relevant national and institutional committees on human experimentation and with the Helsinki Declaration of 1975, as revised in 2008. The registration number for the ethical application and approval 2010‐1185‐31‐5. The register linkage is funded by the Swedish Research Council with grant number 523‐2010‐1052 (Christina Dalman as PI).

## ROLE OF THE FUNDING SOURCE

The funder of the study had no role in study design, data collection, data analysis, data interpretation, or writing of the article. The corresponding author had full access to all the data in the study and had final responsibility for the decision to submit for publication.

## Supporting information

Supporting Information S1

## Data Availability

The data that support the findings of this study are available on request from the corresponding author, EA, under certain circumstances. The data are not publicly available due to ethical restrictions.
