# Supplementary material for: Educational level and the risk of mental disorders, substance use disorders and self‐harm in different age‐groups: A cohort study covering 1,6 million subjects in the Stockholm region
Source: Int J Methods Psychiatr Res. 2023 Feb 17;32(4):e1964. doi: 10.1002/mpr.1964 (PMC10698815; doi:10.1002/mpr.1964)
Supplement: Supplementary file 1 — Supporting Information S1 [file MPR-32-e1964-s001.docx]

**Table 1.** Characteristics of males with a diagnosis of mental disorder, substance use disorders and self-harm during 2001 to 2016 and educational attainment in 2000 by four different age groups

|  | **10-18 years** | | **19-27 years** | | **28-50 years** | | **51-70 years** | |
| --- | --- | --- | --- | --- | --- | --- | --- | --- |
| **Total** | n=147,211 | | n=145, 179 | | n=348,179 | | n=186, 910 | |
| **Diagnoses** | **n*** | **% **** | **n*** | **%**** | **n*** | **%**** | **n*** | **%**** |
| Schizophrenia | 494 | 0.34 | 577 | 0.40 | 2020 | 0.58 | 713 | 0.38 |
| High | \| 235 \| 0.30% \| \| --- \| --- \| | 0.30 | 264 | 0.36 | 384 | 0.27 | 170 | 0.28 |
| Middle | 197 | 0.34 | 203 | 0.35 | 1,024 | 0.67 | 279 | 0.36 |
| Low | 62 | 0.57 | 110 | 0.76 | 612 | 1.18 | 264 | 0.54 |
| Major depression | 13,437 | 9.13 | 13,702 | 9.44 | 34,480 | 9.90 | 14,549 | 7.78 |
| High | 6,583 | 8.45 | 6,311 | 8.65 | 11,693 | 8.12 | 4,835 | 7.90 |
| Middle | 5,741 | 9.84 | 5,792 | 10.02 | 16,246 | 10.67 | 6,091 | 7.89 |
| Low | 1,113 | 10.16 | 1,599 | 11.10 | 6,541 | 12.59 | 3,623 | 7.47 |
| Dysthymia | 482 | 0.33 | 512 | 0.35 | 1,365 | 0.39 | 297 | 0.16 |
| High | 255 | 0.33 | 252 | 0.35 | 465 | 0.32 | 107 | 0.17 |
| Middle | 196 | 0,34 | 208 | 0.36 | 646 | 0.42 | 130 | 0.17 |
| Low | 31 | 0.28 | 52 | 0.36 | 254 | 0.49 | 60 | 0.12 |
| Bipolar disorder | 1162 | 0.79 | 1,176 | 0.81 | 2,762 | 0.79 | 1,015 | 0.54 |
| High | 638 | 0.82 | 596 | 0.82 | 1,005 | 0.70 | 432 | 0.71 |
| Middle | 449 | 0.77 | 449 | 0.78 | 1,319 | 0.87 | 394 | 0.51 |
| Low | 75 | 0.68 | 131 | 0.91 | 438 | 0.84 | 189 | 0.39 |
| Anxiety disorders | 21,944 | 14.91 | 21,940 | 15.11 | 49,059 | 14.09 | 13,916 | 7.45 |
| High | 10,486 | 13.46 | 9,826 | 13.47 | 16,805 | 11.67 | 4,443 | 7.26 |
| Middle | 9,463 | 16.22 | 9,443 | 16.33 | 23,300 | 15.30 | 5,949 | 7.71 |
| Low | 1,995 | 18.21 | 2,671 | 18.55 | 8,954 | 17.24 | 3,524 | 7.26 |
| Anorexia nervosa | 51 | 0.03 | 21 | 0.01 | 13 | 0.00 | 10 | 0.01 |
| High | 29 | 0.04 | 11 | 0.02 | ≤4 | 0.00 | ≤4 | 0.00 |
| Middle | 19 | 0.03 | 6 | 0.01 | 5 | 0.00 | 4 | 0.01 |
| Low | ≤4 | 0.03 | 4 | 0.03 | 5 | 0.01 | ≤4 | 0.01 |
| Bulimia nervosa | 52 | 0.04 | 36 | 0.02 | 46 | 0.01 | 9 | 0.00 |
| High | 21 | 0.03 | 19 | 0.03 | 11 | 0.01 | 4 | 0.01 |
| Middle | 28 | 0.05 | 9 | 0.02 | 28 | 0.02 | ≤4 | 0.00 |
| Low | ≤4 | 0.03 | 8 | 0.06 | 7 | 0.01 | ≤4 | 0.01 |
| Autism spectrum | 2,105 | 1.43 | 1,114 | 0.77 | 1,456 | 0.42 | 103 | 0.06 |
| High | 1,092 | 1.40 | 535 | 0.73 | 419 | 0.29 | 34 | 0.06 |
| Middle | 877 | 1.50 | 468 | 0.81 | 651 | 0.43 | 43 | 0.06 |
| Low | 136 | 1.24 | 111 | 0.77 | 386 | 0.74 | 26 | 0.05 |
| ADHD | 4,546 | 3.09 | 3,090 | 2.13 | 3,823 | 1.1 | 150 | 0.08 |
| High | 1937 | 2.49 | 1,201 | 1.65 | 769 | 0.53 | 51 | 0.08 |
| Middle | 2185 | 3.75 | 1,451 | 2.51 | 2,022 | 1.33 | 65 | 0.08 |
| Low | 424 | 3.87 | 438 | 3.04 | 1,032 | 1.99 | 34 | 0.07 |
| Conduct disorders | 242 | 0.16 | 116 | 0.08 | 204 | 0.06 | 93 | 0.05 |
| High | 97 | 0.12 | 48 | 0.07 | 43 | 0.03 | 25 | 0.04 |
| Middle | 119 | 0.20 | 56 | 0.10 | 96 | 0.06 | 39 | 0.05 |
| Low | 26 | 0.24 | 12 | 0.08 | 65 | 0.13 | 29 | 0.06 |
| Alcohol use dis | 7,592 | 5.16 | 5,183 | 3.57 | 18,445 | 5.30 | 11,245 | 6.02 |
| High | 3160 | 4.06 | 1939 | 2.66 | 4,068 | 2.83 | 2,679 | 4.38 |
| Middle | 3649 | 6.25 | 2,466 | 4.27 | 9,636 | 6.33 | 5,015 | 6.50 |
| Low | 783 | 7.15 | 778 | 5.4 | 4,741 | 9.13 | 3,551 | 7.32 |
| Drug use disorder | 5,938 | 4.03 | 4,013 | 2.76 | 11,010 | 3.16 | 5,761 | 3.08 |
| High | 2,152 | 2.76 | 1,318 | 1.81 | 1,827 | 1.27 | 1,192 | 1.95 |
| Middle | 2,945 | 5.05 | 1,953 | 3.38 | 5,583 | 3.67 | 2,547 | 3.30 |
| Low | 841 | 7.68 | 742 | 5.15 | 3,600 | 6.93 | 2,022 | 4.17 |
| Self-harm | 823 | 0.56 | 635 | 0.44 | 1,225 | 0.35 | 353 | 0.19 |
| High | 317 | 0.41 | 208 | 0.29 | 218 | 0.15 | 83 | 0.14 |
| Middle | 407 | 0.70 | 317 | 0.55 | 627 | 0.41 | 168 | 0.22 |
| Low | 99 | 0.90 | 110 | 0.76 | 380 | 0.73 | 102 | 0.21 |

*Occurrence, e.g., each diagnose is only present once, first time diagnosis for each condition, although the same person can be diagnosed with several conditions. **Percentage of the diagnose at each educational level (among all participants at the same educational level)

**Table 2.** Characteristics of females with a diagnosis of mental disorder, substance use disorders, and self-harm during 2001 and 2016 to educational attainment in 2000 by four different age groups

|  | **10-18 years** | | **19-27 years** | | **28-50 years** | | **51-70 years** | |
| --- | --- | --- | --- | --- | --- | --- | --- | --- |
| **Total** | n= 152, 651 | | n= 144, 801 | | n= 335, 743 | | n= 193, 878 | |
| **Diagnoses** | **n*** | **%**** | **n*** | **%**** | **n*** | **%**** | **n*** | **%**** |
| Schizophrenia | 257 | 0.17 | 341 | 0.24 | 1,754 | 0.52 | 987 | 0.51 |
| High | 125 | 0.16 | 154 | 0.21 | 464 | 0.32 | 231 | 0.35 |
| Middle | 106 | 0.17 | 144 | 0.25 | 851 | 0.57 | 428 | 0.54 |
| Low | 26 | 0.23 | 43 | 0.30 | 439 | 1.11 | 328 | 0.68 |
| Major depression | 25,292 | 16,57 | 25,197 | 17.40 | 59,876 | 17.83 | 27,200 | 14.03 |
| High | 12,413 | 15.51 | 11,539 | 16.09 | 22,739 | 15.52 | 9,141 | 13.72 |
| Middle | 10,861 | 17.71 | 10,738 | 18.35 | 28,048 | 18.75 | 11,269 | 14.32 |
| Low | 2,018 | 17.89 | 2,920 | 20.08 | 9,089 | 22.89 | 6,790 | 13.99 |
| Dysthymia | 713 | 0.47 | 618 | 0.43 | 1,750 | 0.52 | 473 | 0.24 |
| High | 376 | 0.47 | 285 | 0.40 | 584 | 0,40 | 187 | 0.28 |
| Middle | 287 | 0.47 | 263 | 0.45 | 860 | 0.58 | 193 | 0.25 |
| Low | 50 | 0.47 | 70 | 0.48 | 306 | 0.77 | 93 | 0.19 |
| Bipolar disorder | 2,256 | 1.48 | 1,988 | 1.37 | 3,957 | 1.18 | 1,411 | 0.73 |
| High | 1,137 | 1.42 | 929 | 1.30 | 1,542 | 1.05 | 614 | 0.92 |
| Middle | 977 | 1.59 | 857 | 1.46 | 1,852 | 1.24 | 560 | 0.71 |
| Low | 142 | 1.26 | 202 | 1.39 | 563 | 1.42 | 237 | 0.49 |
| Anxiety disorders | 42,517 | 27.85 | 41,455 | 28.63 | 95,843 | 28.55 | 31,715 | 16.36 |
| High | 20,718 | 25.89 | 19,046 | 26.55 | 37,900 | 25.88 | 10,874 | 16.32 |
| Middle | 18,295 | 29.83 | 17, 605 | 30.08 | 45,009 | 30,09 | 13,212 | 16.79 |
| Low | 3,504 | 31.07 | 4,804 | 33.03 | 12,934 | 32,57 | 7,629 | 15.72 |
| Anorexia nervosa | 1,117 | 0.73 | 349 | 0.24 | 245 | 0.07 | 45 | 0.02 |
| High | 696 | 0.87 | 189 | 0.26 | 88 | 0.06 | 17 | 0.03 |
| Middle | 377 | 0.61 | 127 | 0.22 | 115 | 0.08 | 20 | 0.03 |
| Low | 44 | 0.39 | 33 | 0.23 | 42 | 0.11 | 8 | 0.02 |
| Bulimia nervosa | 1,309 | 0.86 | 715 | 0.49 | 472 | 0.14 | 33 | 0.02 |
| High | 744 | 0.93 | 314 | 0.44 | 159 | 0.11 | 16 | 0.02 |
| Middle | 495 | 0.81 | 323 | 0.55 | 240 | 0.16 | 14 | 0.02 |
| Low | 70 | 0.62 | 78 | 0.54 | 73 | 0.18 | ≤4 | 0.01 |
| Autism spectrum | 1,426 | 0.93 | 782 | 0.54 | 1006 | 0.30 | 68 | 0.04 |
| High | 695 | 0.87 | 365 | 0.51 | 292 | 0.20 | 17 | 0.03 |
| Middle | 639 | 1.04 | 331 | 0.57 | 494 | 0.33 | 24 | 0.03 |
| Low | 92 | 0.82 | 86 | 0.59 | 220 | 0.55 | 27 | 0.06 |
| ADHD | 3,989 | 2.61 | 2,733 | 1.89 | 3,437 | 1.02 | 136 | 0.07 |
| High | 1,684 | 2.10 | 1,097 | 1.53 | 888 | 0.61 | 58 | 0.09 |
| Middle | 1,947 | 3.17 | 1,276 | 2.18 | 1,791 | 1,20 | 59 | 0.07 |
| Low | 358 | 3.17 | 360 | 2.48 | 758 | 1.91 | 19 | 0.04 |
| Conduct disorders | 231 | 0.15 | 88 | 0.06 | 208 | 0.06 | 98 | 0.05 |
| High | 105 | 0.13 | 41 | 0.06 | 56 | 0.04 | 31 | 0.05 |
| Middle | 100 | 0.16 | 36 | 0.06 | 100 | 0.07 | 36 | 0.05 |
| Low | 26 | 0.23 | 11 | 0.08 | 52 | 0.13 | 31 | 0.06 |
| Alcohol use dis | 5,961 | 3.90 | 2,804 | 1.94 | 9,692 | 2.89 | 5,122 | 2.64 |
| High | 2,528 | 3.16 | 1,069 | 1.49 | 2,633 | 1.80 | 1,398 | 2.1 |
| Middle | 2,888 | 4.71 | 1,377 | 2.35 | 5,150 | 3.44 | 2,344 | 2.98 |
| Low | 545 | 4.83 | 358 | 2.46 | 1,909 | 4.81 | 1,380 | 2.84 |
| Drug use disorder | 3,846 | 2.52 | 2,916 | 2.01 | 11.359 | 3.38 | 7,030 | 3,63 |
| High | 1,411 | 1.76 | 975 | 1.36 | 2,380 | 1.62 | 1,636 | 2.46 |
| Middle | 1,954 | 3.19 | 1,460 | 2.49 | 6,159 | 4.12 | 3,253 | 4.13 |
| Low | 481 | 4.27 | 481 | 3.31 | 2,820 | 7.10 | 2,141 | 4.14 |
| Self-harm | 1,523 | 1.00 | 795 | 0.55 | 1,500 | 0.45 | 408 | 0.21 |
| High | 671 | 0.84 | 288 | 0.40 | 350 | 0.24 | 127 | 0.19 |
| Middle | 679 | 1.11 | 386 | 0.66 | 786 | 0.53 | 176 | 0.22 |
| Low | 173 | 1.53 | 121 | 0.83 | 364 | 0.92 | 105 | 0.22 |

*Occurrence, e.g., each diagnose is only present once, first time diagnosis for each condition, although the same person can be diagnosed with several conditions.

**Percentage of the diagnose at each educational level (among all participants at the same educational level.
